# Nebulization of the acidified sodium nitrite formulation attenuates acute hypoxic pulmonary vasoconstriction

**DOI:** 10.1186/1465-9921-11-81

**Published:** 2010-06-21

**Authors:** Bakytbek Egemnazarov, Ralph T Schermuly, Bhola K Dahal, Garry T Elliott, Niel C Hoglen, Mark W Surber, Norbert Weissmann, Friedrich Grimminger, Werner Seeger, Hossein A Ghofrani

**Affiliations:** 1 Medical Clinic II, University Hospital Giessen and Marburg, Giessen, Germany; 2 Max-Planck-Institute for Heart and Lung Research, Bad Nauheim, Germany; 3 Aires Pharmaceuticals Inc., San Diego, CA, USA

## Abstract

**Background:**

Generalized hypoxic pulmonary vasoconstriction (HPV) occurring during exposure to hypoxia is a detrimental process resulting in an increase in lung vascular resistance. Nebulization of sodium nitrite has been shown to inhibit HPV. The aim of this project was to investigate and compare the effects of nebulization of nitrite and different formulations of acidified sodium nitrite on acute HPV.

**Methods:**

*Ex vivo *isolated rabbit lungs perfused with erythrocytes in Krebs-Henseleit buffer (adjusted to 10% hematocrit) and *in vivo *anesthetized catheterized rabbits were challenged with periods of hypoxic ventilation alternating with periods of normoxic ventilation. After baseline hypoxic challenges, vehicle, sodium nitrite or acidified sodium nitrite was delivered via nebulization. In the *ex vivo *model, pulmonary arterial pressure and nitric oxide concentrations in exhaled gas were monitored. Nitrite and nitrite/nitrate were measured in samples of perfusion buffer. Pulmonary arterial pressure, systemic arterial pressure, cardiac output and blood gases were monitored in the *in vivo *model.

**Results:**

In the *ex vivo *model, nitrite nebulization attenuated HPV and increased nitric oxide concentrations in exhaled gas and nitrite concentrations in the perfusate. The acidified forms of sodium nitrite induced higher levels of nitric oxide in exhaled gas and had longer vasodilating effects compared to nitrite alone. All nitrite formulations increased concentrations of circulating nitrite to the same degree. In the *in vivo *model, inhaled nitrite inhibited HPV, while pulmonary arterial pressure, cardiac output and blood gases were not affected. All nitrite formulations had similar potency to inhibit HPV. The tested concentration of appeared tolerable.

**Conclusion:**

Nitrite alone and in acidified forms effectively and similarly attenuates HPV. However, acidified nitrite formulations induce a more pronounced increase in nitric oxide exhalation.

## Background

Hypoxia-induced pulmonary arterial vasoconstriction is an important physiologic mechanism leading to redistribution of blood flow from poorly ventilated areas of the lung to better ventilated ones in an attempt to optimize ventilation-perfusion matching [1]. Generalized HPV, which occurs during exposure to hypoxia or at high altitudes, is a pathophysiologic process resulting in an acute increase in pulmonary vascular resistance, right ventricular overload, and restriction of right ventricular function [2]. Chronic hypoxic exposure, which accompanies lung diseases such as chronic obstructive pulmonary disease and pulmonary arterial hypertension, results in sustained HPV and vascular remodeling, which in turn leads to acceleration of right ventricular failure [3]. Therefore, resolution of HPV is a viable strategy for treatment of these patients.

NO is a potent endothelial-derived vasodilating agent [4]. Inhaled administration of exogenous NO has a vasodilatory effect that is selective for the pulmonary vasculature [5]. Moreover, activation of the NO-cGMP signaling pathway has been shown to attenuate HPV [6] and has antiproliferative effects [7].

Recently, nitrite metabolism has gained attention because it may represent an endogenous store of NO [8]. Enzymes having nitrite reductase activity include deoxyhemoglobin [9] and xanthine oxidoreductase [10]. Non-enzymatic NO generation from nitrite under acidic conditions [11] in the stomach or due to reaction with ascorbic acid has also been demonstrated [12,13]. Further, nitrite reduction to NO in a physiologically acidic environment (pH 6.66) has been shown to relax isolated aortic rings. This effect was augmented in the presence of ascorbic acid [14].

However, the hemodynamic importance of these mechanisms is still subject of debate because the NO-producing activity of deoxyhemoglobin can be overwhelmed by the NO-scavenging properties of hemoglobin [15]. On the other hand, nitrite delivered to the lungs of newborn sheep by nebulization induced NO release and inhibited HPV [16]. Moreover, increased blood flow after nitrite infusion has been demonstrated in humans [17,18]. Topical administration of acidified nitrite formulations has been shown to increase blood flow in skin [19].

Thus, mammals have both nitrite reducing systems to promote vasodilation and systems capable of blunting this vasodilating effect. The aim of this study was to test whether application of acidified sodium nitrite formulations could inhibit HPV in an *ex vivo *and *in vivo *rabbit model and if there are differences in the effects of nitrite alone and acidified formulations. Additionally, we sought to test the possibility of restricting the vasodilating effect of nitrite to the pulmonary vasculature by nebulization of the nitrite formulations [20].

## Methods

### Chemicals and reagents

Krebs-Henseleit buffer contained 120 mM NaCl, 4.3 mM KCl, 1.1 mM KH_2_PO_4_, 24 mM NaHCO_3_, 2.4 mM CaCl_2_, 1.3 mM MgCl_2_, and 13.32 mM glucose as well as 5% (wt/vol) hydroxyethylamylopectin (mol wt 200,000) and was purchased from Serag-Wiessner (Naila, Germany). Sodium nitrite, citric acid, ascorbic acid and saccharin were obtained from Aires Pharmaceuticals (San Diego, USA). Griess reagent (modified) and vanadium chloride were obtained from Sigma (Munich, Germany).

### Animals

All animal experiments were approved by local authorities. Male New Zealand White Rabbits (body weight 3.33 ± 0.24 kg) were used. Animals were kept under pathogen-free conditions and handled in accordance with the European Communities recommendations for experimentation.

### Isolated lung model

The technique of isolated rabbit lung perfusion has been described [21]. Briefly, rabbits were anticoagulated intravenous administration of heparin 1,000 U/kg and deeply anesthetized with ketamine and xylazine. The lungs were removed from the thorax without interrupting ventilation and perfused and freely suspended from a force transducer for monitoring organ weight in a humidified chamber at 39°C. In a recirculating system, the flow was slowly increased to 100 ml/min (total volume 250 ml). Left atrial pressure was set at 2 mmHg (referenced at the hilum), and the entire system was equilibrated at 37°C.

**Figure 1:**
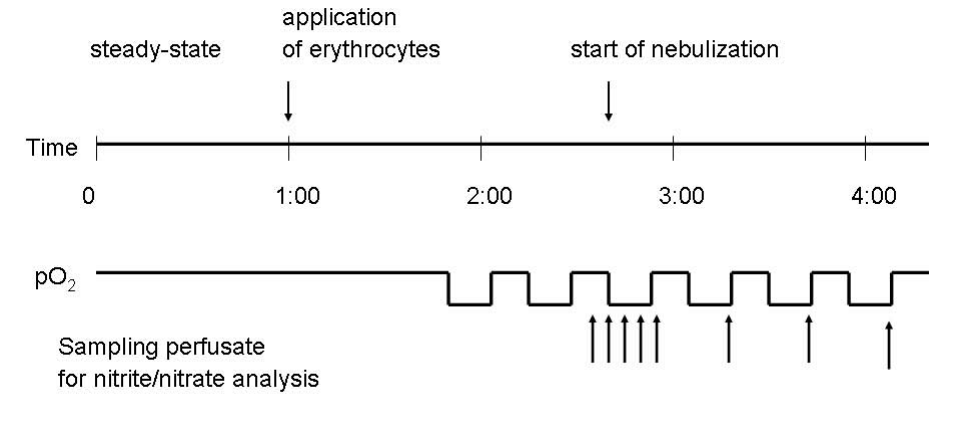
**Timescale of *ex vivo *experimental setup**. A total of six hypoxic maneuvers (3% O_2 _in an inhaled gas mixture) were performed. The second hypoxic maneuver was used as a control, because the second and subsequent hypoxic maneuvers induced HPV of stable and reproducible strength. Nebulization was initiated at the onset of the third hypoxic maneuver. In dose titration experiments, nitrite nebulizations were performed beginning with the third maneuver. The concentration that was delivered increased with each subsequent nebulization.

Pressures in the pulmonary artery and left atrium were registered with pressure transducers, and data were continuously transferred to a computer for online monitoring. Inclusion criteria for the study were *1*) a homogeneous white appearance of the lungs with no signs of edema, hemostasis or atelectasis; *2*) initial Ppa and ventilation pressure values in the normal range; and *3*) a constant organ weight during an initial steady-state period of at least 20 minutes. Figure 1 illustrates the basic design of the experiments.

### Erythrocyte isolation

During lung preparation, blood was collected by puncturing the left ventricle. After centrifugation for 10 minutes at 300 × *g*, the buffy coat was removed. The remaining erythrocytes were washed by mixing with Krebs-Henseleit buffer (1:1 ratio) and re-centrifuged. Supernatant was removed, and sedimented erythrocytes were admixed with re-circulating buffer according to the protocol (see Figure 1).

### Experimental protocol

After the initial steady-state period, washed autologous erythrocytes were admixed with recirculating buffer to achieve a level of 10% hematocrit. A blood transfusion filter (Pall, Dreieich, Germany) was used to remove erythrocyte aggregates from circulation. After a 50-minute stabilization period, hypoxic challenges were initiated by switching the inhaled gas mixture to a hypoxic mixture of 3% O_2_, 5.3% CO_2_, and 94.7% N_2 _(Messer, Siegen, Germany). In dose-escalation experiments, after the first two hypoxic maneuvers every hypoxic challenge was accompanied by nebulization of sodium nitrite in increasing concentrations. Dosages tested were 1 μg/ml, 10 μg/ml, 100 μg/ml, 1 mg/ml, 5 mg/ml and 10 mg/ml. Dosages were calculated in ALF.

The experimental protocol for testing different formulations is presented in Figure 1. Six sequential hypoxic maneuvers of 15 minutes duration interrupted by 15-minute periods of normoxic ventilation were conducted. During the third hypoxic challenge, nebulization was carried out using the AeronedLab nebulizer system (Respironics, Herrsching, Germany) either with PB (0.05 mM) or different sodium nitrite formulations dissolved in PB.

Lungs were treated with one of the following formulations:

1. PB: 1 ml 0.05 mM PB. The pH of the solution was 7.8-7.9;

2. Nitrite: 1 ml of the 0.05 mM PB containing 220 mM sodium nitrite and 0.25 mM sodium saccharin. The pH of the solution was 7.7-7.8;

3. Nitrite + citric acid: 1 ml of 0.05 mM PB containing 220 mM sodium nitrite, 1.56 mM citric acid, and 0.25 mM sodium saccharin. The pH of the solution was 5.4-5.5;

4. Nitrite + citric acid + ascorbic acid: 1 ml of 0.05 mM PB containing 220 mM sodium nitrite, 1.56 mM citric acid, 0.5 mM ascorbic acid, and 0.25 mM sodium saccharin. The pH of the solution was 5.4-5.5.

Nebulization of these formulations of sodium nitrite resulted in nitrite concentrations in ALF of approximately 1 mg/ml. This concentration is based on the volume of ALF in our experimental setup, found to be 2.5 ml [22], and the delivery efficiency of our nebulization system at approximately 25%, estimated by weighing the nebulizer system before and after nebulization.

### Measurement of Noex

Measurements were performed as described by Spriestersbach *et al*. [23]. Briefly, aliquots of exhaled gas were taken continuously for measurement by chemiluminescence using a Sievers 280 NO Analyzer (Seeheim/Ober-Beerbach, Germany).

### Measurement of nitrite and nitrate in perfusate

Nitrite in the perfusate samples was measured using Griess reagent according to manufacturer's instructions (Sigma, Munich, Germany). Briefly, perfusate was sampled from venous effluent at the times illustrated in Scheme 1. After centrifugation, the supernatant was aliquoted, frozen immediately, and stored at -20°C until analysis. For measurement of nitrite, 100 μl of perfusate was mixed with 100 μl of Griess reagent. After 15-minute incubation at room temperature, the absorbance at 540 nm was measured. The nitrite concentration in experimental samples was calculated by comparing values against a calibration curve. Calibration curve was generated by serial dilutions of sodium nitrite of known concentrations in the same buffer as used for experiments. To measure nitrite and nitrate levels, samples were incubated with Griess reagent for 10 minutes, and then 100 μl vanadium chloride was added. After 35-minute incubation at 36°C, the absorbance was measured.

### Wet to dry ratio determination

At the completion of perfusion experiments, pieces of lung tissue from every lobe were cut. Samples were weighed and dried in an oven at 60°C for 7-9 days until the weight stabilized. Initial weight (wet) was divided by final weight (dry).

### Rabbit surgical preparation

Techniques for the measurement of invasive hemodynamics are described elsewhere [24]. Briefly, rabbits were anesthetized with xylazine (2.1 mg/kg) and ketamine (7 mg/kg), followed by a constant intravenous infusion of xylazine (25 mg/kg/h) and ketamine (80 mg/kg/h) (Injectomat S; Fresenius, Bad Hamburg, Germany) through the right peripheral ear vein. Rabbits were anticoagulated with heparin (200 U/kg), tracheostomized and ventilated with an FI_O2 _of 0.5 using a Harvard respirator (cat/rabbit ventilator; Hugo Sachs Elektronik, March Hugstetten, Germany). Frequency was set at 40 breaths/min, tidal volume at 8 ml/kg, and PEEP at 0.5 mmHg resulting in a paCO_2 _range of 35-45 mmHg. A catheter was inserted into the left carotid artery and connected to a pressure transducer for arterial pressure monitoring. The right femoral vein was cannulated for infusion of saline. A balloon-tipped pulmonary artery catheter (Berman angiographic balloon catheter AI-07134, 4F; Arrow, Reading, PA) was inserted into the pulmonary artery through the right external jugular vein.

### Hemodynamics and blood gases

Ppa and mean Psa were continuously recorded using fluid-filled pressure transducers (Braun; Combitrans, Melsungen, Germany). The level of the left atrium was the zero reference for measurements. CO was calculated by the Fick's principle, employing the mixed venous oxygen content, arterial oxygen content, and oxygen uptake. Oxygen uptake of the animals was measured online (O_2 _controller; Labotect, Göttingen, Germany). Arterial and mixed venous samples were collected (1 ml) and maintained on ice until analyzed for pO_2_, pH, and pCO_2 _(ABL330; Radiometer, Copenhagen, Denmark). Hemoglobin and oxygen saturation were measured using an OSM2 hemoximeter (Radiometer).

### Experimental protocol

After the initial steady-state period, hypoxic challenges were initiated by reducing the FI_O2 _to 0.15. Six sequential hypoxic maneuvers of 15 minutes duration were interrupted by 15-minute periods of normoxic ventilation. During the third hypoxic challenge, nebulization of either PB (0.05 mM) or different sodium nitrite formulations dissolved in PB was performed. Blood samples for gas analysis were drawn during the steady-state period and during hypoxic challenges with ongoing nebulization.

### Data analysis

Data are expressed as mean ± s.e.m. For multiple comparisons, one-way analysis of variance followed by a post-hoc test (Student-Newman-Keuls) was used. To evaluate intra-group differences, a paired t-test was used. *P *values < 0.05 were considered significant.

## Results

### Nitrite nebulization reduces HPV in the rabbit *ex vivo *model

**Figure 2:**
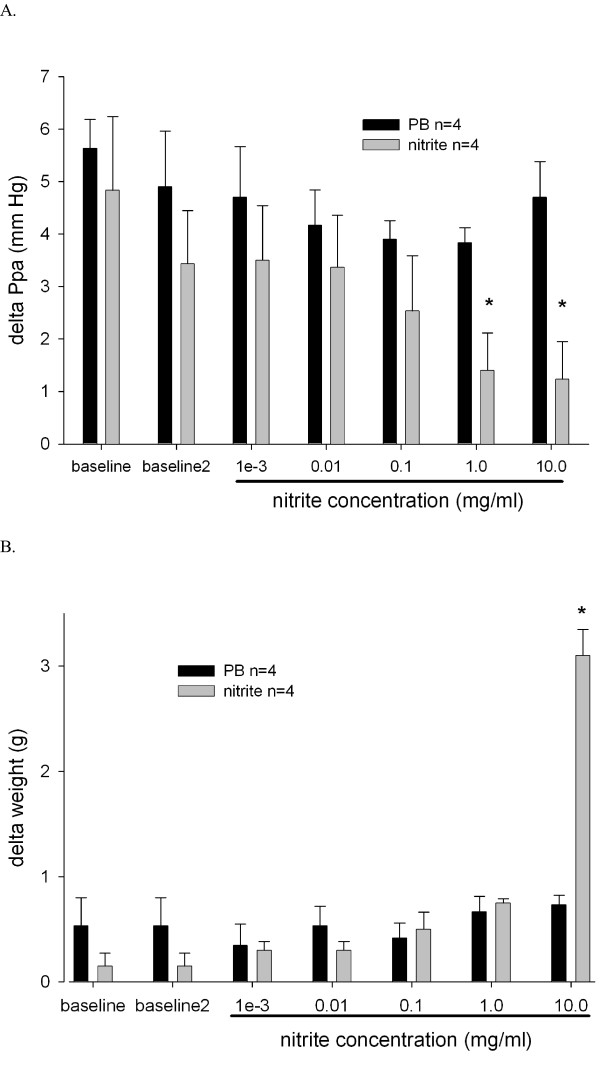
**Dose titration for nebulized nitrite in the isolated perfused lung model**. Nitrite was nebulized in increasing concentrations during hypoxic maneuvers. **(A) **HPV was calculated as the difference between the maximum Ppa during hypoxia and the Ppa during normoxia. **(B) **Lung weight gain during nebulization. Lungs were freely suspended from a force transducer for monitoring organ weight in a humidified chamber at 39°C. Weight changes were monitored continuously. Values represent differences in lung weight before and after each nebulization procedure. * p < 0.05 vs. PB.

**Figure 3:**
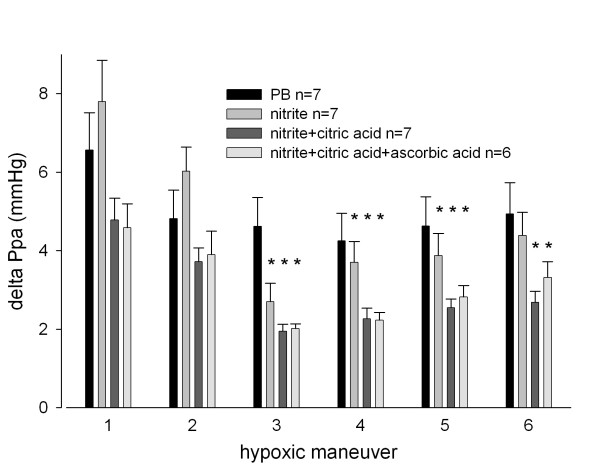
**Effect of nebulized nitrite and acidified forms of nitrite on the strength of HPV in the isolated perfused lung model**. The strength of HPV is represented as the difference between the maximum Ppa during hypoxia and the Ppa during normoxia. * p < 0.05 vs. PB.

Ventilation with 15-minute periods of hypoxia induced acute pulmonary vasoconstriction (Figure 2, 3). All hypoxic challenges, except the first, induced reproducible vasoconstriction. Therefore, the second HPV response was used as a reference. Nebulization of sodium nitrite or vehicle was performed during the third hypoxic challenge, and experiments were complete after the sixth hypoxic challenge. Initial experiments were performed to determine the concentration of sodium nitrite needed for a significant change in Ppa. In these dose escalation experiments, sodium nitrite at both 1 and 10 mg/ml in ALF was found to reduce the HPV response (Figure 2A). However, nitrite at 10 mg/ml induced a significant increase in lung weight during nebulization, suggesting toxicity (Figure 2B) and therefore a dose of 1 mg/ml was chosen for the remaining experiments. Sodium nitrite effectively reduced the strength of HPV, whereas nebulization with PB had no discernable effect on HPV (Figure 3).

Acidified preparations appeared to have a more pronounced effect on Ppa compared to sodium nitrite alone. Importantly, a single nebulization of nitrite reduced the strength of HPV by 50.9% ± 8.8%. This effect lasted for three hypoxic challenges. These results indicated that sodium nitrite in acidified preparations may have a more sustained effect on HPV than sodium nitrate alone (Figure 3) as evidenced by significant reductions throughout the last hypoxic challenge (*i.e*., the entire length of the study).

**Figure 4:**
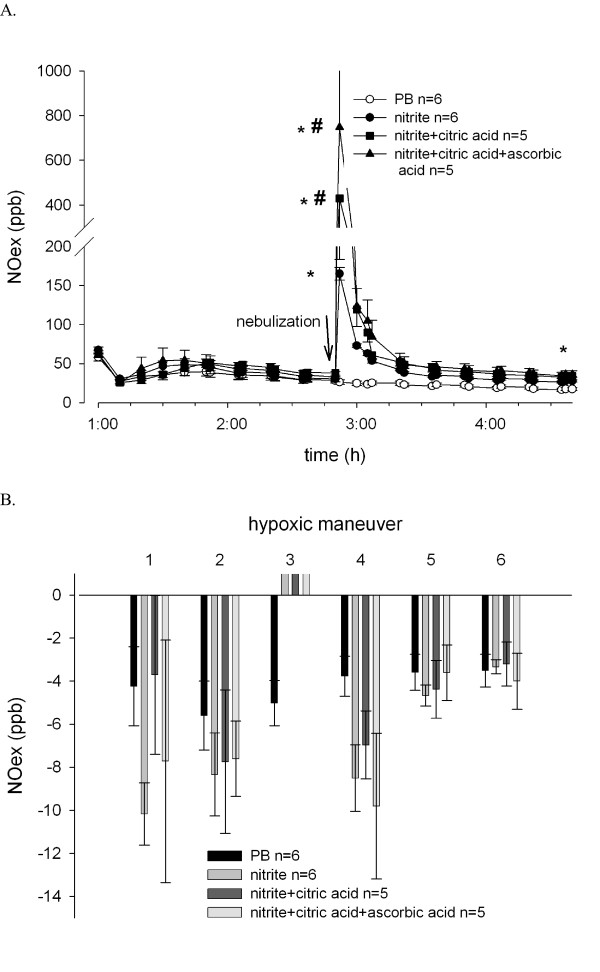
**Impact of nitrite nebulization on the concentration of exhaled NO in the isolated perfused lung model**. **(A) **Exhaled NO was monitored online from the exhaled gas mixture. **(B) **Hypoxic challenges induced a rapid and reversible decrease in NOex. Nitrite nebulization increased NOex during the hypoxic phase. Values represent the difference between NOex concentrations during hypoxia and during the preceding normoxic phase. * p < 0.05 vs. PB, # p < 0.05 vs. nitrite.

NOex was monitored after nebulization of the various sodium nitrite preparations. Nitrite nebulization significantly increased the concentration of NOex during nebulization. The combination of sodium nitrite with citric acid or ascorbic acid induced a more pronounced increase in the concentration of NOex compared to sodium nitrite alone (Figure 3A). NOex concentrations remained elevated in all nitrite nebulization groups until termination of the experiment (nitrite group 27.9 ± 1.3 ppb and PB group 17.4 ± 2.1 ppb at the end of experiment, p = 0.009). However, during the hypoxic maneuvers, changes in NOex were similar in all groups (Figure 4B).

**Figure 5:**
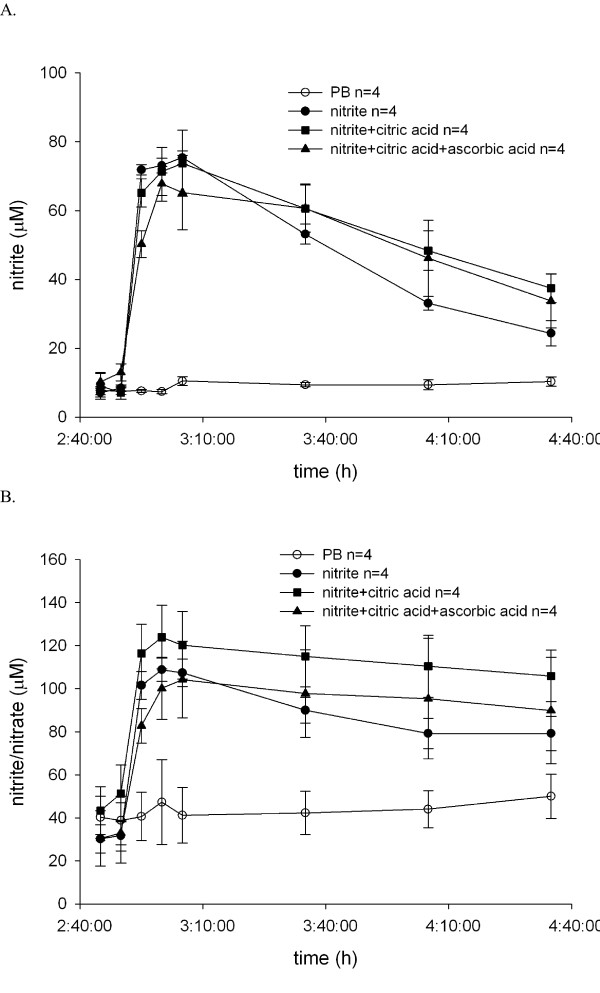
**Changes in nitrite and nitrite/nitrate (NOx) concentration in perfusate after nebulization**. Perfusate samples were collected as depicted in Scheme 1. Samples were immediately frozen and stored at -20°C until analysis. Measurements were performed using a modified Griess reagent according to manufacturer's instructions. **(A) **Changes in nitrite concentration in recirculating perfusate. **(B) **Changes in NOx concentration in perfusate.

**Figure 6:**
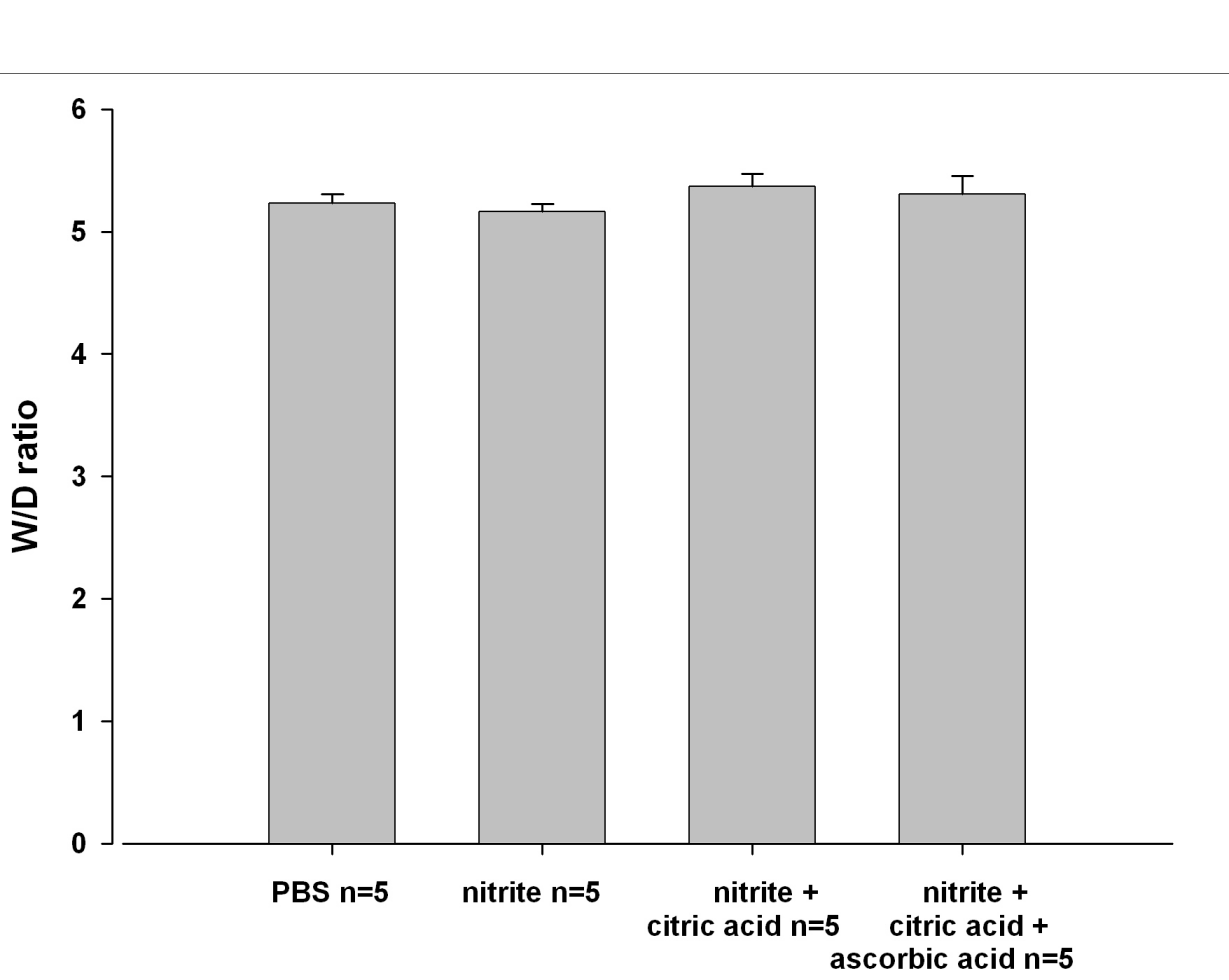
**Impact of nitrite nebulization on lung wet to dry ratio**. At the end of each experiment, pieces of lung tissue from every lobe were cut. Samples were weighed and dried in an oven at 60°C for 7-9 days until the weight stabilized. Initial weight (wet) was divided by final (dry) weight.

Nitrite nebulization resulted in a rapid increase in nitrite and nitrate concentrations in recirculating buffer (Figure 5), whereas PB nebulization did not. Different formulations of sodium nitrite increased the nitrite level in perfusion buffer to the same extent. Nebulization of sodium nitrite from these formulations did not alter fluid retention in lungs, as assessed by the absence of changes in the wet to dry ratio (Figure 6).

### Nitrite nebulization reduces HPV *in vivo*

A second series of experiments was performed in lungs of live rabbits. All rabbits demonstrated stable hemodynamics during experiments without the need for support with adrenomimetics. We chose a level of hypoxia that induced pronounced HPV without significantly changing Psa. Hypoxic maneuvers induced a rapid decrease in paO_2 _from 260.7 ± 3.6 mmHg to 45.1 ± 1.8 mmHg. Results were similar in all groups. SaO_2 _decreased from 99.7 ± 0.01% to 86.5 ± 1.6%. This induced acute and reversible HPV (Table 1), which was accompanied by a slight decrease in Psa. Nebulization of sodium nitrite and its acidified forms effectively reduced HPV to one-third of its initial value (Table 1). In groups receiving nebulized sodium nitrite, the strength of HPV during subsequent hypoxic challenges remained slightly reduced.

**Table 1:** The effect of nebulized nitrite and acidified forms of nitrite on the strength of HPV, Psa and CO in the *in vivo *model of catheterized rabbits.

		PB	PB + nitrite	PB + nitrite + citrate	p value
HPV	Baseline (mmHg)	1.24 ± 0.58	1.56 ± 0.38	1.79 ± 0.50	n.s.
	Nebulization (mmHg)	1.36 ± 0.50	0.52 ± 0.39	0.69 ± 0.72	p < 0.05
	(% of baseline value)	119.52 ± 36.74	32.28 ± 26.13	36.30 ± 51.32	p < 0.05

Systemic hypoxic hypotension	Baseline (mmHg)	-6.78 ± 1.97	-6.07 ± 0.64	-7.39 ± 2.61	n.s.
	Nebulization (mmHg)	-6.36 ± 1.27	-5.28 ± 1.39	-8.16 ± 0.85	n.s.

Cardiac output	Baseline (ml/min)	617.63 ± 15.96	725.64 ± 39.59	575.46 ± 34.64	n.s.
	Nebulization (ml/min)	556.79 ± 37.62	589.46 ± 33.28	533.05 ± 28.16	n.s.

HPV was calculated as the difference between the maximum Ppa during hypoxia and the Ppa during normoxia. For percentage calculations, the strength of HPV during the second hypoxic challenge was set to 100%, and the value of HPV during nebulization was related to it. Nitrite alone and in acidified forms nebulized during the third hypoxic maneuver significantly inhibited HPV. CO was measured by Fick's method. Blood samples for analysis were drawn during normoxia and at the end of the third hypoxic challenge. Psa was monitored continuously using a catheter placed in the left carotid artery. Values represent the difference between Psa during normoxia and Psa during subsequent hypoxia.

Notably, nitrite nebulization did not influence Psa and did not augment hypoxia-induced decreases in Psa. However, acidified nitrite slightly, but not significantly, augmented hypoxia-induced decreases in Psa during the third hypoxic maneuver (Table 1). CO was comparable in all groups. Sodium nitrite nebulization did not affect CO (Table 1).

## Discussion

We sought to test whether inhalation of acidified sodium nitrite formulations could reduce the extent of hypoxia-induced vasoconstriction. Further, we examined whether acidified formulations had more pronounced vasodilatory effects than nitrite alone as a result of the synergistic action of two different nitrite-reducing mechanisms. The main finding of our study is that both nebulized nitrite alone and acidified formulations effectively reduced the strength of HPV in both *ex vivo *and *in vivo *models of acute HPV.

Hunters *et al*. observed increased NOex and reduced HPV after nitrite nebulization in lambs [16]. These data suggest that inhaled application of nitrite induces preferential vasodilation in the pulmonary circulation, operating via deoxyhemoglobin oxidation by nitrite with NO generation, a mechanism described by Doyle *et al*. [25]. However, Deem *et al*. demonstrated that, although this mechanism is operational during hypoxia, its effect is overwhelmed by the NO scavenging property of hemoglobin [15]. These findings suggest limited efficacy of the mechanism of nitrite reduction by deoxyhemoglobin for the treatment of patients. On the other hand, nitrite reductase activity of other enzymes expressed in the lung, for instance xanthine oxidase and mitochondrial respiratory chain, has been described [26]. Combined, these findings could represent an important mechanism responsible for nitrite-induced vasodilation.

Recently, a non-enzymatic pathway of NO generation, resulting from nitrite dysproportionation under acidic pH during anoxia, has gained attention [27]. The highest yield of NO production in anoxic myocardium was found to occur at pH 5.5 [28]. Moreover, topical administration of acidified nitrite formulations induced local vasodilation, presumably resulting from enhanced NO generation [19]. NO generation at acidic pH was potentiated in the presence of ascorbic acid, which led to greater vasodilation.

In accordance with the findings of other investigators, we observed that nebulization of nitrite to ventilated rabbit lungs during hypoxic challenge significantly reduced HPV. Inhibition of vasoconstriction was associated with increased NOex during nitrite nebulization.

The combination of nitrite and citric acid or citric acid and ascorbic acid resulted in acidic formulations with pH values of 5.3-5.4. This pH range was chosen to achieve the maximal rate of non-enzymatic NO generation. The acidification of nitrite solutions led to spontaneous NO generation *in vitro *(data not shown), in accordance with the mechanism of nitrite dysproportionation described by Zweier *et al*. [27]. Nebulization of the acidified nitrite formulations to isolated lungs induced a more pronounced increase in NOex than the solution of nitrite alone. Further, acidified formulations induced longer-lasting vasodilation than nitrite alone, although they reduced HPV to the same degree as sodium nitrite. Notably, nitrite nebulization induced a dramatic increase in NOex lasting for 10 minutes. Levels of NOex remained elevated until the termination of the experiment in all nitrite nebulization groups.

Nebulization of nitrite led to a rapid and sustained increase in nitrite concentration in recirculating buffer. The maximum concentration of nitrite in the buffer was 71.3 ± 7.0 μM, which is within the range of effective vasodilating concentrations of nitrite described by others [14,29,30]. Higher concentrations of circulating nitrite (as achieved by nebulization) induced lung weight gain. The physiological significance of the elevated level of nitrite in the buffer is not clear. It could represent a depot that delivers and releases nitrite on demand. Conversely, according to the mechanism described by Doyle *et al*. [25], subsequent hypoxic challenges should lead to deoxygenation with deoxyhemoglobin formation, a process that converts nitrite to NO. Indeed, we observed that in all subsequent hypoxic challenges HPV was slightly lower, although this reduction did not reach statistical significance. However, enhanced NOex, as a result of reaction between sodium nitrite and deoxyhemoglobin during hypoxic maneuvers, was not observed (Figure 3B).

Using the 1 mg/ml formulations, significant fluid accumulation in the lung after nitrite nebulization was not observed, suggesting that nitrite in the concentration used in this study had no adverse effect on the lung.

In the *in vivo *experiments, sodium nitrite nebulization effectively reduced the strength of HPV in the first hypoxic challenge following nebulization. The effect of both sodium nitrite alone and acidified nitrite formulations lasted for only 15 minutes, although HPV remained slightly reduced during subsequent hypoxic challenges until termination of the experiment. CO did not change significantly during the nitrite nebulization experiments. In contrast to intravenous injection of nitrite, which induces both pulmonary and systemic vasodilation [31], nebulized sodium nitrite induced vasodilation in the lung with no appreciable effect on systemic hemodynamics. However, nebulization of acidified formulations of nitrite slightly augmented hypoxic vasodilation of systemic vessels.

In summary, we observed higher levels of NOex in experiments involving acidified nitrite nebulization. However, we did not observe further enhancement of the HPV-inhibiting effect. Notably, we observed slightly more systemic vasodilation in the acidified nitrite group. However, this effect did not reach statistical significance. Nevertheless, this could be an important consideration in patients suffering from pulmonary hypertension who have limited cardiac reserve and borderline systemic hypotension. One possible explanation is that acidified nitrite is not as dependant as non-acidified nitrite from the local microenvironment and deoxyhemoglobin formation. Therefore, the effects of acidified nitrite formulations may be not restricted to the hypoxic areas of the lung.

We used the *ex vivo *model of isolated perfused rabbit lung for dose calibration and pharmacokinetic studies. The limitation of this model is that pharmacokinetic and pharmacodynamic data obtained in this manner do not reflect the influence of metabolism and excretion by kidney and liver. Indeed, in our *in vivo *model we observed a shorter duration of the inhibitory effects of nebulized nitrite on HPV. This may reflect different pharmacokinetics *in vivo*. Nevertheless, our results obtained from the *in vivo *model demonstrate that the mechanism described is operational *in vivo *and has physiological relevance.

## Conclusions

In the isolated lung preparation, both of the acidified nitrite formulations we used, as well as nitrite alone, effectively and similarly attenuated HPV with a trend toward a more sustained effect with acidified formulations. NOex increased when nitrite was prepared in an acidic buffer, suggesting a potential means for better efficacy. The concentrations of nitrite we tested appeared tolerable. Nebulization of the nitrite or acidic nitrite formulations preferentially induced vasodilation of the pulmonary vasculature with no appreciable effect on systemic arterial pressure or cardiac output *in vivo*.

## List of used abbreviations

ALF: alveolar lining fluid; PB: phosphate buffer; CO: cardiac output; FI_O2_: fraction of inspired oxygen; HPV: hypoxic pulmonary vasoconstriction; NO: nitric oxide; NOex: exhaled nitric oxide; NOx: nitrate and nitrite; Ppa: pulmonary arterial pressure; Psa: systemic arterial pressure; SaO_2_: oxygen saturation of hemoglobin; PEEP: positive end-expiratory pressure.

## Competing interests

BE, RTS, BKD, NW, FG, WS, HAG - these authors declare no conflict of interest. GTE, NCH, MWS - declare that they are employees of Aires Pharmaceuticals Inc.

## Authors' contributions

BE, RTS, BKD, GTE, NCH, MWS NW, FG, WS, HAG contributed to the conception and design of the study. BE, RTS, BKD, MWS performed experiments, evaluated results, and interpreted data. BE, RTS, BKD, GTE, NCH, MWS NW, FG, WS, HAG were involved in interpretation of data. BE, RTS, BKD, GTE, NCH, MWS NW, FG, WS, HAG were involved in drafting and revising the manuscript for important intellectual content. GTE, HAG gave final approval of the version to be published. All authors read and approved the final manuscript.

## References

[B1] Moudgil R Michelakis ED Archer SL Hypoxic pulmonary vasoconstriction J Appl Physiol 2005 98 1 390 403 10.1152/japplphysiol.00733.2004 15591309

[B2] Wagner PD Reduced maximal cardiac output at altitude--mechanisms and significance Respir Physiol 2000 120 1 1 11 10.1016/S0034-5687(99)00101-2 10786640

[B3] Wright JL Levy RD Churg A Pulmonary hypertension in chronic obstructive pulmonary disease: current theories of pathogenesis and their implications for treatment Thorax 2005 60 7 605 609 10.1136/thx.2005.042994 15994270 PMC1747459

[B4] Adnot S Raffestin B Eddahibi S NO in the lung Respir Physiol 1995 101 2 109 120 10.1016/0034-5687(95)00016-7 8570913

[B5] Frostell C Fratacci MD Wain JC Jones R Zapol WM Inhaled nitric oxide. A selective pulmonary vasodilator reversing hypoxic pulmonary vasoconstriction Circulation 1991 83 6 2038 2047 2040056 10.1161/01.cir.83.6.2038

[B6] Weissmann N Voswinckel R Tadic A Hardebusch T Ghofrani HA Schermuly RT Seeger W Grimminger F Nitric oxide (NO)-dependent but not NO-independent guanylate cyclase activation attenuates hypoxic vasoconstriction in rabbit lungs Am J Respir Cell Mol Biol 2000 23 2 222 227 10919989 10.1165/ajrcmb.23.2.3935

[B7] Dumitrascu R Weissmann N Ghofrani HA Dony E Beuerlein K Schmidt H Stasch JP Gnoth MJ Seeger W Grimminger F Schermuly RT Activation of soluble guanylate cyclase reverses experimental pulmonary hypertension and vascular remodeling Circulation 2006 113 2 286 295 10.1161/CIRCULATIONAHA.105.581405 16391154

[B8] Lundberg JO Weitzberg E Gladwin MT The nitrate-nitrite-nitric oxide pathway in physiology and therapeutics Nat Rev Drug Discov 2008 7 2 156 167 10.1038/nrd2466 18167491

[B9] Cosby K Partovi KS Crawford JH Patel RP Reiter CD Martyr S Yang BK Waclawiw MA Zalos G Xu X Huang KT Shields H Kim-Shapiro DB Schechter AN Cannon RO Gladwin MT Nitrite reduction to nitric oxide by deoxyhemoglobin vasodilates the human circulation Nat Med 2003 9 12 1498 1505 10.1038/nm954 14595407

[B10] Millar TM Stevens CR Benjamin N Eisenthal R Harrison R Blake DR Xanthine oxidoreductase catalyses the reduction of nitrates and nitrite to nitric oxide under hypoxic conditions FEBS Lett 1998 427 2 225 228 10.1016/S0014-5793(98)00430-X 9607316

[B11] Zweier JL Samouilov A Kuppusamy P Non-enzymatic nitric oxide synthesis in biological systems Biochimica et Biophysica Acta (BBA) - Bioenergetics 1999 1411 2-3 250 10.1016/S0005-2728(99)00018-3 10320661

[B12] Mirvish SS Wallcave L Eagen M Shubik P Ascorbate-nitrite reaction: possible means of blocking the formation of carcinogenic N-nitroso compounds Science 1972 177 43 65 68 10.1126/science.177.4043.65 5041776

[B13] May JM How does ascorbic acid prevent endothelial dysfunction? Free Radical Biology and Medicine 2000 28 9 1421 10.1016/S0891-5849(00)00269-0 10924860

[B14] Modin A Bjorne H Herulf M Alving K Weitzberg E Lundberg JO Nitrite-derived nitric oxide: a possible mediator of 'acidic-metabolic' vasodilation Acta Physiol Scand 2001 171 1 9 16 10.1046/j.1365-201x.2001.171001009.x 11350258

[B15] Deem S Min JH Moulding JD Eveland R Swenson ER Red blood cells prevent inhibition of hypoxic pulmonary vasoconstriction by nitrite in isolated, perfused rat lungs Am J Physiol Heart Circ Physiol 2007 292 2 H963 970 10.1152/ajpheart.00812.2006 17012349

[B16] Hunter CJ Dejam A Blood AB Shields H Kim-Shapiro DB Machado RF Tarekegn S Mulla N Hopper AO Schechter AN Power GG Gladwin MT Inhaled nebulized nitrite is a hypoxia-sensitive NO-dependent selective pulmonary vasodilator Nat Med 2004 10 10 1122 1127 10.1038/nm1109 15361865

[B17] Dejam A Hunter CJ Tremonti C Pluta RM Hon YY Grimes G Partovi K Pelletier MM Oldfield EH Cannon RO III Schechter AN Gladwin MT Nitrite Infusion in Humans and Nonhuman Primates: Endocrine Effects, Pharmacokinetics, and Tolerance Formation Circulation 2007 116 16 1821 1831 10.1161/CIRCULATIONAHA.107.712133 17893272

[B18] Maher AR Milsom AB Gunaruwan P Abozguia K Ahmed I Weaver RA Thomas P Ashrafian H Born GV James PE Frenneaux MP Hypoxic modulation of exogenous nitrite-induced vasodilation in humans Circulation 2008 117 5 670 677 10.1161/CIRCULATIONAHA.107.719591 18212289

[B19] Gribbe Ö Gustafsson LE Wiklund NP Transdermally administered nitric oxide by application of acidified nitrite increases blood flow in rat epigastric island skin flaps European Journal of Pharmacology 2008 578 1 51 10.1016/j.ejphar.2007.09.032 17976572

[B20] Brilli RJ Krafte-Jacobs B Smith DJ Passerini D Moore L Ballard ET Aerosolization of novel nitric oxide donors selectively reduce pulmonary hypertension Crit Care Med 1998 26 8 1390 1396 10.1097/00003246-199808000-00026 9710099

[B21] Seeger W Menger M Walmrath D Becker G Grimminger F Neuhof H Arachidonic acid lipoxygenase pathways and increased vascular permeability in isolated rabbit lungs Am Rev Respir Dis 1987 136 4 964 972 2821856 10.1164/ajrccm/136.4.964

[B22] Ghofrani HA Kohstall MG Weissmann N Schmehl T Schermuly RT Seeger W Grimminger F Alveolar epithelial barrier functions in ventilated perfused rabbit lungs Am J Physiol Lung Cell Mol Physiol 2001 280 5 L896 904 11290513 10.1152/ajplung.2001.280.5.L896

[B23] Spriestersbach R Grimminger F Weissmann N Walmrath D Seeger W On-line measurement of nitric oxide generation in buffer-perfused rabbit lungs J Appl Physiol 1995 78 4 1502 1508 7615462 10.1152/jappl.1995.78.4.1502

[B24] Schermuly RT Ghofrani HA Enke B Weissmann N Grimminger F Seeger W Schudt C Walmrath D Low-dose systemic phosphodiesterase inhibitors amplify the pulmonary vasodilatory response to inhaled prostacyclin in experimental pulmonary hypertension Am J Respir Crit Care Med 1999 160 5 1500 1506 10556112 10.1164/ajrccm.160.5.9901102

[B25] Doyle MP Pickering RA DeWeert TM Hoekstra JW Pater D Kinetics and mechanism of the oxidation of human deoxyhemoglobin by nitrites J Biol Chem 1981 256 23 12393 12398 7298665

[B26] van Faassen EE Bahrami S Feelisch M Hogg N Kelm M Kim-Shapiro DB Kozlov AV Li H Lundberg JO Mason R Nohl H Rassaf T Samouilov A Slama-Schwok A Shiva S Vanin AF Weitzberg E Zweier J Gladwin MT Nitrite as regulator of hypoxic signaling in mammalian physiology Med Res Rev 2009 29 5 683 741 10.1002/med.20151 19219851 PMC2725214

[B27] Zweier JL Wang P Samouilov A Kuppusamy P Enzyme-independent formation of nitric oxide in biological tissues Nat Med 1995 1 8 804 809 10.1038/nm0895-804 7585184

[B28] Zweier JL Samouilov A Kuppusamy P Non-enzymatic nitric oxide synthesis in biological systems Biochim Biophys Acta 1999 1411 2-3 250 262 10.1016/S0005-2728(99)00018-3 10320661

[B29] Dalsgaard T Simonsen U Fago A Nitrite-dependent vasodilation is facilitated by hypoxia and is independent of known NO-generating nitrite reductase activities Am J Physiol Heart Circ Physiol 2007 292 6 H3072 3078 10.1152/ajpheart.01298.2006 17307993

[B30] Crawford JH Isbell TS Huang Z Shiva S Chacko BK Schechter AN Darley-Usmar VM Kerby JD Lang JD Jr Kraus D Ho C Gladwin MT Patel RP Hypoxia, red blood cells, and nitrite regulate NO-dependent hypoxic vasodilation Blood 2006 107 2 566 574 10.1182/blood-2005-07-2668 16195332 PMC1895612

[B31] Casey DB Badejo AM Jr Dhaliwal JS Murthy SN Hyman AL Nossaman BD Kadowitz PJ Pulmonary vasodilator responses to sodium nitrite are mediated by an allopurinol-sensitive mechanism in the rat Am J Physiol Heart Circ Physiol 2009 296 2 H524 533 10.1152/ajpheart.00543.2008 19074675 PMC2643888

